# Quercetin-Imprinted Nanospheres as Novel Drug Delivery Devices

**DOI:** 10.3390/jfb3020269

**Published:** 2012-03-29

**Authors:** Manuela Curcio, Giuseppe Cirillo, Ortensia Ilaria Parisi, Francesca Iemma, Nevio Picci, Francesco Puoci

**Affiliations:** Department of Pharmaceutical Sciences, University of Calabria, Arcavacata di Rende (CS) 87036, Italy; Email: giuseppe.cirillo@unical.it (G.C.); ortensiailaria.parisi@unical.it (O.I.P.); francesca.iemma@unical.it (F.I.); nevio.picci@unical.it (N.P.); francesco.puoci@unical.it (F.P.)

**Keywords:** molecularly imprinted polymers, quercetin, drug delivery, cytotoxicity test, nanomaterials

## Abstract

In this work, molecularly imprinted nanospheres for controlled/sustained release of quercetin were synthesized employing methacrylic acid and ethylene glycoldymethacrylate as functional monomer and crosslinking agent, respectively. One pot precipitation polymerization was chosen as polymerization technique to obtain nanosized materials with spherical shape. Morphological and hydrophilic properties by scanning electron microscopy and water content measurements were determined, and recognition and selectivity properties of the imprinted materials were tested using the template quercetin and its structural analogue, the flavonoid catechin. Finally, the applicability of the obtained materials as drug delivery devices was evaluated by performing *in vitro* release studies in plasma simulating fluids and cytotoxicity testson HeLa cells.

## 1. Introduction

Nanotechnology and nanoparticulate carriers offer unique promises in many biomedical fields, such as in disease prevention, diagnosis and controlled drug delivery [1]. The advantages in using this kind of materials in drug delivery lie in the possibility of obtaining a target-specific delivery of drugs and genes to various sites in the body, in the increase of the drug residence at the target site and in the improvement of cellular uptake and intracellular stability [2].

Furthermore, it is well known that the enhanced permeability and retention (EPR) effect of the tumor vasculature allows the nanoparticulate carriers to preferentially accumulate in cancer tissue and to release the loaded drug in the target site [3]. In this way it is also possible to reduce the side-effects of many bioactives used in several pathological states, such as in cancer therapy, maximizing their therapeutic potential. Since several chemotherapeutic agents are small hydrophobic molecules with poor solubility, they are quickly cleared through the blood stream upon intravenous injection [4]. Thus, their incorporation in nanoparticulate carriers is an optimum strategy to enhance their concentration in the target site [5].

Recently, the coupling of the properties of nanoparticulate and hydrogel systems within molecular imprinting strategy has attracted considerable research interest because of the wide potential applications of imprinted nanomaterials in bioassays and in the synthesis of drug delivery systems [6,7,8,9]. Molecular imprinting is a very useful and straightforward method to impart a molecular memory to a polymer network [10]. In brief, the target molecule, named template, is entrapped in a pre-polymerization complex constituted by one or more suitable functional monomers that interact with the functionalities of the template by covalent or non-covalent interactions. Subsequently, the addition of a crosslinking agent to the polymerization mixture “freezes” this complex in a tridimensional network. Finally, the removal of the template leaves in the polymer structure binding cavities that maintain the size and shape of the template molecule, conferring high selectivity to the polymer network.

In the last two decades, Molecular Imprinted Polymers (MIP) have found application in a wide number of research areas, such as in chromatographic separations and solid-phase extraction as stationary phases [11], catalysis [12], sensors [13], artificial antibody [14] and controlled drug delivery devices [15].

The employment of MIP as drug carriers offers two main potential advantages: comparing to the traditional polymeric devices, indeed, this technology provides materials with higher drug loading capacity and an additional level of control of drug release profile, due to the presence of the binding sites that interact more strongly with the target molecule, allowing a considerable improvements of the device properties [6,16].

An important requirement of the materials which have to be used in drug delivery is a considerable degree of flexibility. Hydrogel systems are soft materials which well respond to this necessity, because the swelling capacity confers them high flexibility and makes them very similar to living tissues. To date, only a small number of research articles report on the application of imprinted materials in drug delivery, and this is mainly due to the difficulty in imparting the molecular memory to materials with a significant flexibility of the polymer chains [17,18]. The majority of imprinted polymers, indeed, are characterized by high crosslinking degree in order to limit the mobility of the associated binding cavities and obtain materials with better selectivity properties.

Recently, attempts have been made to obtain nano-sized imprinted particulates to be used in drug delivery. Ciardelli *et al*. [19] prepared theophylline imprinted nanospheres by precipitation polymerization using methyl methacrylate and methacrylic acid (MAA) as functional monomers and trimethylolpropanetrimethacrylate (TRIM) as crosslinker, while, in a more recent work [20], dipyridamole imprinted nanospheres were synthesized by employing the same polymerization technique and using MAA and TRIM as functional monomer and crosslinking agent, respectively. Binding experiments performed in aqueous buffer and in a complex environment of human serum showed a good imprinting effect, while the more controlled release profile for MIP materials, in comparison with NIP, demonstrated a potential applicability of the proposed materials as drug delivery devices.

This work reports on the synthesis and characterization of imprinted nanospheres with high swelling properties obtained using quercetin (QC), MAA and ethylene glycol dimethacrylate (EGDMA) as template, functional monomer and crosslinking agent, respectively.

**Figure 1:**
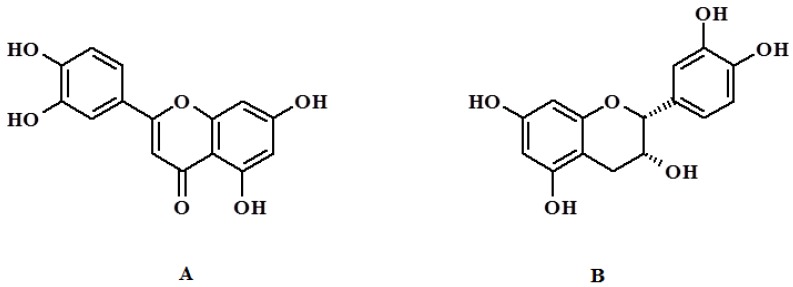
Chemical structure of quercetin (QC) (**A**) and CT (**B**).

QC (3,3',4',5,7-pentahydroxyflavone, Figure 1) is a plant-derived flavonoid commonly found in many foods, including apples, tea, onions, nuts, berries, cauliflower, and cabbage. It possesses many biological properties, such as antioxidant, anti-inflammatory, and anticancer properties [21,22,23].

The main limitation in therapeutic employment of quercetin is its poor solubility in water and instability in physiological medium. This disadvantage restricts the use of this flavonoid to oral administration [24]. Many research works report on the synthesis of nanoparticulate systems to be used as device for the controlled release of QC [25,26,27], but no example of molecular imprinted spherical nanoparticles for systemic drug delivery is described.

The purpose of this study was to investigate the possibility of employing these monodispersed imprinted nanoparticles as devices for the controlled/sustained release of QC. Moreover, cytotoxicity tests were conducted on HeLa cancer cells.

## 2. Results and Discussion

### 2.1. Synthesis and Characterization of QC Imprinted Polymers

**Figure 2:**
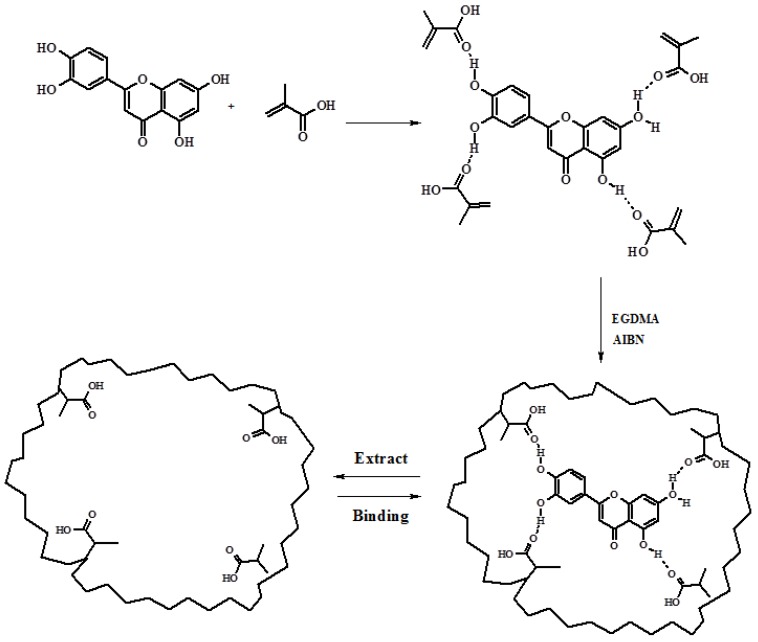
Schematic representation of Molecular Imprinted Polymers (MIP) for QC.

QC imprinted nanospheres were synthesized exploiting the non-covalent imprinting approach using MAA as functional monomer, able to interact with the template by hydrogen bonds, and EGDMA as crosslinking agent(Figure 2).

Non-covalent approach is still the most widely used method to prepare MIP, because of the possibility to mime the interactions in biological systems (especially when the interactions between template molecule and polymeric matrices were based on H-bonds), and the fast equilibrium kinetics.

Furthermore, in order to obtain monodispersed spherical systems in the submicron scale, precipitation polymerization as synthetic methodology was chosen. This is probably the most facile synthetic procedure that provides MIP microspheres and nanospheres with a good control of the morphology and, recently, many research works report on the employment of this technique to obtain monodispersed microgels/nanogels in the size range of 10–300 nm [28,29]. In comparison to materials with irregular shape, spherical systems can provide isotropic release behaviour and so a better control of drug release profile. In addition, in the imprinted nano-sized materials, the enhanced specific surface area can contribute to make the binding sites more accessible to the template.

**Figure 3:**
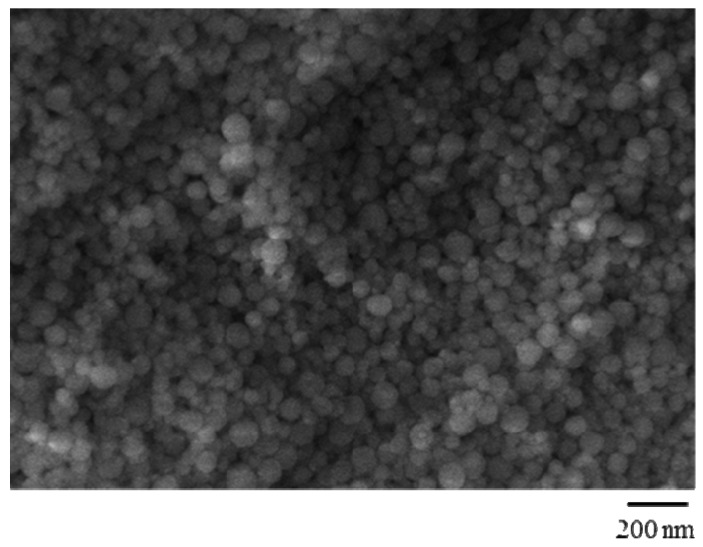
Scanning Electron Micrograph analysis on imprinted nanospheres.

Spherical geometry and the practically monodispersion of prepared samples were confirmed by Scanning Electron Micrographs (Figure 3) and dimensional analysis.

In Table 1, polymerization feed composition and mean particle sizes (d_n_) of the nanospheres are reported.

**Table 1:** Polymerization feed composition and mean particle sizes (d_n_) of MIP and corresponding non-imprinted polymer (NIP) as means ± S.D.

Polymers	QC/MAA/EGDMA (mmol)	d_n_ (nm)	Polydispersivity
MIP	1.0/16.0/25.0	83	1.08
NIP	--/16.0/25.0	87	1.06

The imprinted polymers to be used in drug delivery should possess a certain degree of flexibility, in order to minimize potential irritation to surrounding tissues and obtain a fast equilibrium between the release and re-uptake of the template in the cavity, but also maintain the conformation of the imprinted cavities also in absence of the template, to preserve their selectivity properties [30].

By performing swelling experiments in phosphate buffer at pH 7.4 at different times (from 1 to 24 h), an enhancement of the swelling percentages of MIP and NIP was recorded in the first 2 h (Table 2), then they remained almost constant until 24 h.

**Table 2:** QC and CT bound percentages by imprinted and non-imprinted nanospheres after 2 h in ethanol:water (5:5 v/v) mixture, and water content (%) of polymeric matrices at pH 7.4. Data are shown as means ± S.D.

Matrix	% Q Bound	%CT Bound	α_Q_	α_CT_	Ε	Water Content (%)
MIP	25 ± 1.1	14 ± 1.4	2.27	1.2	1.78	558 ± 0.6
NIP	11 ± 1.3	11 ± 1.1				535 ± 0.5

These high swelling characteristics result very favourable for the polymer performances for two main reasons: the first one is due to the fact that soft materials possess good biocompatibility properties and are suitable for biological applications; the second is ascribable to the enhancement of the recognition properties of the imprinted nanospheres, because the binding sites result much more accessible to the template in swellable than in rigid matrices.

### 2.2. Evaluation of the Imprinting Effect: Binding Experiments in Water Media

The imprinting effect of nanospheres was evaluated by binding experiments in which amounts of polymeric particles (20 mg) were incubated for 2 h with a 0.2 mM standard solution of QC in an etanol:water (5:5 v/v) mixture. In Table 2, the QC binding percentages by MIP and NIP, and three different parameters, α_Q_, α_CT_ and ε, highlighting the imprinting effect of the synthesized materials, are reported [31].

The higher Q binding percentage of MIP, comparing to NIP, demonstrated the presence of binding sites in its structure. This result is interesting also considering that the binding medium contains water, which notably can interfere with the recognition properties of the imprinted polymer, due of the enhancement of the non-specific hydrophobically driven bonds between template and the surface of polymeric materials [32], and can be ascribable to the relative hydrophilicity of the polymeric matrices. The parameter α_Q_ is a measure of the recognition properties of the imprinted materials and was determined as the ratio between the amount (%) of QC bound by MIP and NIP, respectively [33]; this value (2.27) clearly proves the specificity of the interaction between the template and the functional groups on the polymeric nanoparticles.

To evaluate the selectivity of imprinted polymers, the same binding experiments were performed using CT, another flavonoid possessing a structure very similar to QC (Figure 1).As reported for QC, α_CT_ was determined as ratio between CT bound by MIP and NIP, respectively. The low value (1.2) shows high chemical and spatial complementarity of MIP binding sites toward the template.

Finally, the last coefficient (ε) is derived from the ratio between the MIP bound percentages towards QC and CT, respectively. This number represent another measure of the selectivity of the nanospheres and were calculated to be 1.79, indicating that imprinted polymers had higher affinity for QC comparing to CT.

### 2.3. *In Vitro* Release Studies

QC was loaded on the imprinted and non-imprinted nanospheres by soaking procedure, employing the same solvent mixture used in the binding experiments, in order to maximize the interactions polymer-template and, thus, also the loading efficiency. After the incubation time, samples were dried under vacuum and release experiments performed. The percentage of released QC was calculated considering that the total amount of the loaded Q was absorbed by the samples [34].

**Figure 4:**
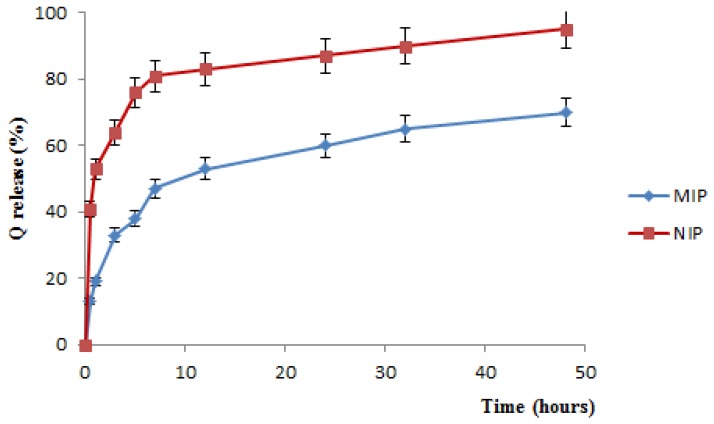
QC release profile from MIP and NIP in plasma simulating fluids.

The Figure 4 shows a considerable difference in the Q release profiles from MIP and NIP. The QC release profile from MIP, indeed, is much more controlled over time than that observed for NIP, and it does not result yet complete even after 48 h.

For NIP materials, moreover, a very marked burst effect, due to the weak interactions between the template and the polymeric matrix, is recorded. Just in the first 30 min, indeed, QC release percentage raised more than 40% of the loaded drug, while for imprinted materials, at the same time, the release percentage is of 13%.

The difference in the QC release profile from MIP and NIP polymers is ascribable to the presence, in imprinted materials, of the binding sites that interact strongly with template, so that a very long time is required to overcome the hydrogen interactions of quercetin molecules with their binding sites, resulting in an extended and controlled release over time.

In the non-imprinted polymers, the flavonoid interacts with the polymer surface by non-covalent interactions. At pH 7.4 the carboxylic groups are ionized, the diffusion rate of the buffer on the polymer surface is fast and the drug is rapidly released. Instead, in the MIP case, the diffusion rate of the buffer into specific cavities of imprinted polymers is slower, and the functional groups are ionized more slowly, resulted in well controlled release [35].

In order to further evaluate selectivity of MIP, CT release experiments in the same conditions tested with Q were also performed. The data obtained from these experiments confirmed results exposed for binding tests: CT release from imprinted nanoparticles, indeed, is much faster and is completed in the first 3 h (data not shown).

### 2.4. Cytotoxicity Test

MTT assay was used as methodology to evaluate the effect of MIP-Q on the viability of HeLa cancer cells, a cell lines widely used to assess the anticancer activity of a tested compound by virtue of their high stability and proliferation properties [36]. MTT assay is an established colorimetric assay for measuring the activity of mitochondrial enzymes present in healthy cells by monitoring the absorbance of purple formazan (570 nm) formed as the enzymatic reduction product of MTT (410 nm).This method is used extensively to evaluate the anti-proliferative effect and the cell viability modulating activity of natural and synthetic compounds [37,38]. Several researchers have documented the ability of QC to modulate signal transduction pathways associated with cell proliferation and apoptosis and to show cytotoxic and antiproliferative effects of quercetin in various human cancer cell lines [39,40,41].

**Figure 5:**
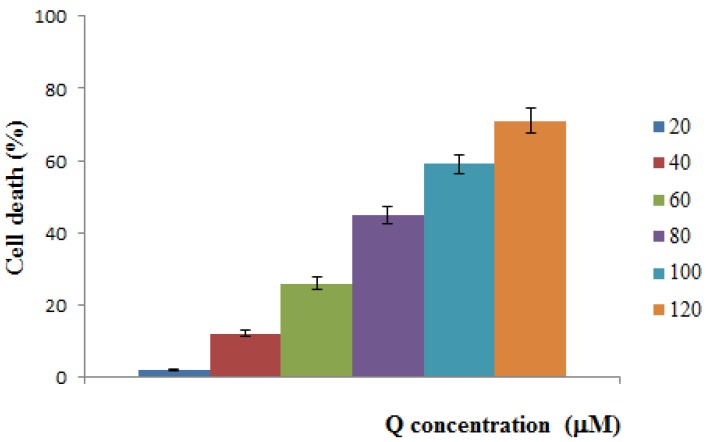
QC inhibitory profile on HeLa cells ± SD.

In this work, a plot experiment was performed to evaluate the QC inhibitory profile on HeLa cancer cells (Figure 5) and the results confirmed the potent antiproliferative activity of QC against the tested cells with an IC_50_ value of 83.4 µM. The anticancer activity of QC loaded into the MIP particles was then tested by using a MIP-QC containing a QC concentration of 100 µM. The so-treated cells were incubated for 48 h and the MTT assay showed that the antiprolifertive activity of QC was preserved after loading into MIP materials, with an inhibition value of 49%.The same experiment, conducted on the non-loaded nanoparticles, showed that the polymeric material does not significantly interfere with the cell viability.

## 3. Experimental Section

### 3.1. Reagents and Standards

MAA, EGDMA, 2,2-azoisobutyronitrile (AIBN), QC and (+)-catechin (CA) were obtained from Sigma–Aldrich (Sigma Chemical Co., St. Louis, MO, USA). All solvents were reagent grade or HPLC-grade and used without further purification and were provided by FlukaChemika-Biochemika (Buchs, Switzerland). MAA was purified by vacuum distillation before use.

### 3.2. Synthesis of QC Imprinted Nanospheres

Imprinted nanospheres were prepared by precipitation polymerization using QC as template, MAA as functional monomer and ethylene glycol dimethacrylate as crosslinking agent. General synthetic procedure was reported: template (1 mmol) and MAA (16 mmol) were dissolved in a mixture of acetonitrile (25 mL) and methanol (25 mL), in a 100 mL round bottom flask and then EGDMA (25 mmol) and AIBN (100 mg) were added. The polymerization mixture was degassed in a sonicating water bath, purged with nitrogen for 10 min cooling with an ice-bath. The flask was then gently agitated (55 rpm) in an oil bath. The temperature was increased from room temperature to 60 °C within 2 h, and then kept at 60 °C for 24 h. At the end of the reaction, the particles were filtered, washed with 100 mL of ethanol, 100 mL of acetone and then with 100 mL of diethyl ether. The template was extracted by ‘‘Soxhlet apparatus’’ using methanol-acetic acid mixture (1:1 (v/v), 100 mL) for at least 48 h, followed by methanol for another 48 h, and monitoring the drug concentration in the extraction solvent by HPLC. Particles were successively dried under vacuum overnight at 40 °C. Blank polymers, acting as a control, were also prepared when polymerization was carried out in the absence of QC.

### 3.3. Water Content of Imprinted Polymers

Aliquots (40–50 mg) of the nanospheres dried to constant weight were placed in a tared 5-mL sintered glass filter (Ø10 mm; porosity, G3), weighted, and left to swell by immersing the filter plus support in a beaker containing the swelling media: phosphate buffer (pH 7.4, simulated biological fluids). At predetermined times (1–2–4–8–10–15–20–24 h), the excess water was removed by percolation at atmospheric pressure. Then, the filter was placed in a properly sized centrifuge test tube by fixing it with the help of a bored silicone stopper, then centrifuged at 3500 rpm for 15 min and weighted. The filter tare was determined after centrifugation with only water. The weights recorded at the different times were averaged and used to give the water content percent (WR%) by the following Equation (1):

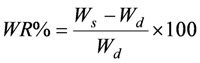
(1)
where W_s_ and W_d_ are weights of swollen and dried spherical microparticles, respectively. Each experiment was carried out in triplicate.

### 3.4. Binding Experiments

Binding experiments were performed in ethanol:water mixture (5:5 v/v). Briefly, 20 mg of polymer particles were mixed with 1 mL QC solution (0.2 mM) in a 1 mL eppendorf and sealed. The tubes were oscillated by a wrist action shaker (Burrell Scientific) in a water bath for 2 h. Then the mixture was centrifuged for 10 min (10,000 rpm) and the QC concentration in the liquid phase was measured by HPLC. The amount of QC bound to the polymer was obtained by comparing its concentration in the imprinted samples to the non-imprinted ones. The same experiments were performed using catechinsolution. Experiments were repeated five times.

### 3.5. Drug Loading by the Soaking Procedure

Polymeric matrix (0.2 g) was immersed in 3 mL of QC solution (6.66 mg/mL) in ethanol:water mixture (5:5 v/v) and soaked at room temperature. During this time, the mixture was continuously stirred and then the solvent was removed by filtration. Finally the powder was dried under vacuum overnight at 40 °C. The same experiments were performed using CT solution.

### 3.6. *In Vitro* Release Studies

Release studies were carried out using the dissolution method described in the USP XXIV (apparatus 1-basket stirring element). The samples (5 mg) were dispersed in flasks containing 10 mL of phosphate buffer (10 mM, pH 7.4), simulating biological fluids, at 37 °C. Thus, at appropriate time intervals, samples were drawn from dissolution medium to determine the amounts of drug released by HPLC. The amount of QC released from six samples of each formulation was used to characterize drug release. The same experiments were performed using particles loaded with CT. Experiments were repeated five times.

### 3.7. Cell Growth Inhibition Studies

The cytotoxicity of QC loaded imprinted nanospheres against human cervical cancer (HeLa) cells was measured by MTT assay, based on the reduction of MTT by mitochondrial dehydrogenases of viable cells to a purple formazon product. Briefly, cells were plated at a density of 1 × 10^5^ cells/mL into 96-well plates and maintained in culture in DMEM supplemented with 10% heat-inactivated FBS, 100 units/mL penicillin and 100 mg/mL streptomycin, at 37 °C in a humid atmosphere containing 5% CO_2_. After overnight growth, cells were treated with 20, 40, 60, 80, 100, 120 and 140 µM concentrations of quercetin for 48 h to record the inhibition profile of the antioxidant. Subsequently, the cells were washed with 200 µL of PBS, and incubated with 100 µL of 500 µg/mL MTT in PBS at 37 °C for 3 h. MTT was removed and DMSO was added to dissolve the formazan crystals [38,41]. The optical density at 570 nm was determined using a BioRadmicroplate reader. Untreated cells were taken as control with 100% viability and cells without addition of MTT were used as blank to calibrate the spectrophotometer to zero absorbance. Triton X-100 1% was used as positive control of cytotoxicity. Experiments were repeated three times. The same experimental protocol was applied to cells treated with QC-loaded MIP (QC equivalent concentration of 140 µM) and unloaded MIP to verify the effect of the polymeric materials on the assay.

### 3.8. HPLC Analysis

A Jasco BIP-I pump and Jasco UVDEC-100-V detector set at 350 nm [42] were used. A 250 × 4 mm C-18 Hibar^®^ column, 10 mm particle size (Merck, Darmstadt, Germany) was employed. The mobile phase was ACN and 0.1% (w/v) H_3_PO_4_ aqueous solution (36:64, v/v). Column oven temperature was controlled at 25 °C. The flow rate was maintained at 0.5 mL min^−1^ and 20 mL of sample was injected.

### 3.9. Scanning Electron Microscopy

Scanning electron microscopy (SEM) photographs were obtained with a Jeol JSMT 300 A; the surface of the samples was made conductive by deposition of a gold layer on the samples in a vacuum chamber.

### 3.10. Dimensional Analysis

Approximate range in particle size was determined by measuring 300 particles per each sample with the use of an image processing and analysis system, a Leica DMRB equipped with a LEICAWild 3D stereomicroscope text paragraph

## 4. Conclusions

This work reports on a preliminary study about the potential applicability of QC imprinted nanospheres as drug delivery devices. Precipitation polymerization was chosen as synthetic methodology to obtain particles with spherical shape, while MAA and EGDMAwere employed in the polymerization feed as functional monomer and crosslinker, respectively. The obtained materials showed high swelling characteristics and good recognition properties in aqueous medium. These ones were demonstrated through binding experiments with template and its analogue, catechin.

The results obtained from the *in vitro* release studies in plasma simulating fluids and the cytotoxicity tests indicated the suitability of these materials as devices for the controlled/sustained delivery of QC in biological fluids. In comparison with non-imprinted materials, certainly the QC release profile from MIP is much more controlled over time due to the presence of the binding sites, while the cytotoxicity assay, performed on HeLa cells, showed not only that the polymeric material does not significantly interfere with the cell viability, but also that the antiproliferative activity of QC was preserved after loading onto MIP materials.

Further attempts should be performed in order to characterize the release kinetics for the short and long term in conditions mimicking physiological environments and to optimize the performances of the imprinted nanospheres in physiological medium.

## References

[1] Couvreur P., Vauthier C. (2007). Nanotechnology: Intelligent design to treat complex disease. Pharm. Res..

[2] Torchilin V.P. (2007). Targeted pharmaceutical nanocarriers for cancer therapy and imaging. AAPS J..

[3] Maeda H., Wu J., Sawa T., Matsumura Y., Hori K. (2000). Tumor vascular permeability and the EPR effect in macromolecular therapeutics: A review. J. Control. Release.

[4] Chinsriwongkul A., Chareanputtakhun P., Ngawhirunpat T., Rojanarata T., Sila-On W., Rutkanonkai U., Opanasopit P. (2012). Nanostructured Lipid Carriers (NLC) for parenteral delivery of ananticancer drug. AAPS PharmSciTech.

[5] Chauhan A.S., Jain N.K., Diwan P.V., Khopade A.J. (2004). Solubility enhancement of indomethacin with withpoly(amidoamine) dendrimersand targeting to inflammatory regions of arthritic rats. J. Drug Target..

[6] Byrne M.E., Salian V. (2004). Molecular imprinting within hydrogels II: Progress and analysis of the field. Int. J. Pharm..

[7] Lu C., Zhou W., Han B., Yang H., Chen X., Wang X. (2007). Surface-imprinted core-shell nanoparticles for sorbent assays. Anal. Chem..

[8] Xie C., Liu B., Wang Z., Gao D., Guan G., Zhang Z. (2008). Molecular imprinting at walls of silica nanotubes for TNT recognition. Anal. Chem..

[9] Cirillo G., Iemma F., Puoci F., Parisi O.I., Curcio M., Spizzirri U.G., Picci N. (2009). Imprinted hydrophilic nanospheres as drug delivery systems for 5-fluorouracil sustained release. J. Drug Target..

[10] Curcio M., Puoci F., Cirillo G., Iemma F., Spizzirri U.G., Picci N. (2010). Selective determination of melamine in aqueous medium by molecularly imprinted solid phase extraction. J. Agric. Food Chem..

[11] Vidyasankar S., Arnold F.H. (1995). Molecular imprinting: selective materials for separations, sensors and catalysis. Curr. Opin. Biotechnol..

[12] Li P., Huang Y., Hu J., Yuan C., Lin B. (2002). Surface plasmon resonance studies on molecular imprinting. Sensors.

[13] Ansell R.J., Ramström O., Mosbach K. (1996). Towards artificial antibodies prepared by molecular imprinting. Clin. Chem..

[14] Cirillo G., Curcio M., Parisi O.I., Puoci F., Iemma F., Spizzirri U.G., Picci N. (2010). Gastro-intestinal sustainedreleaseofphyticacidbymolecularlyimprintedmicroparticles. Pharm. Dev. Technol..

[15] Cunliffe D., Kirby A., Alexander C. (2005). Molecularly imprinted drug delivery systems. Adv. Drug Deliv. Rev..

[16] Sellergren B., Allender C.J. (2005). Molecularly imprinted polymers: A bridge to advanced drug delivery. Adv. Drug Deliv. Rev..

[17] Singh B., Chauhan N., Sharma V. (2011). Design of molecular imprinted hydrogels for controlled release of cisplatin: Evaluation of network density of hydrogels. Ind. Eng. Chem. Res..

[18] Alvarez-Lorenzo C., Yañez F., Barreiro-Iglesias R., Concheiro A. (2006). Imprinted soft contact lenses as norfloxacin delivery systems. J. Control. Release.

[19] Ciardelli G., Cioni B., Cristallini C., Barbani N., Silvestri D., Giusti P. (2004). Acrylic polymeric nanospheres for the release and recognition ofmolecules of clinical interest. Biosens. Bioelectron..

[20] Esfandyari-Manesh M., Javanbakht M., Atyabi F., Mohammadi A., Mohammadi S., Akbari-Adergani B., Dinarvand R. (2011). Dipyridamole recognition and controlled release by uniformly sized molecularly imprinted nanospheres. Mater. Sci. Eng. C.

[21] Adlercreutz H., Mousavi Y., Hockerstedt K. (1992). Diet and breast cancer. Acta Oncol..

[22] Ferry D.R., Smith A., Malkhandi J., Fyfe D.W., Takats P.G., Anderson D., Baker J., Kerr D.J. (1996). Phase I clinical trial of the flavonoid quercetin: Pharmacokinetics and evidence for *in vivo* tyrosine kinase inhibition. Clin. Cancer Res..

[23] Moon Y.J., Wang L., DiCenzo R., Morris M.E. (2008). Quercetin pharmacokinetics in humans. Biopharm. Drug Dispos..

[24] Zheng Y., Haworth I.S., Zuo Z., Chow M.S.S., Chow A.H.L. (2005). Physicochemical and structural characterization of quer-cetin-b-cyclodextrin complexes. J. Pharm. Sci..

[25] Kumari A., Yadav S.K., Pakade Y.B., Singh B., Yadav S.C. (2010). Development of biodegradable nanoparticles for delivery of quercetin. Colloids Surf. B.

[26] Lee D.H., Sim G.S., Kim J.H., Lee G.S., Pyo H.B., Lee B.C. (2007). Preparation and characterization of quercetin-loadedpolymethyl methacrylate microcapsules usinga polyol-in-oil-in-polyol emulsion solventevaporation method. J. Pharm. Pharmacol..

[27] Barreto A.C.H., Santiago V.R., Mazzetto S.E., Denardin J.C., Lavìn R., Mele G., Ribeiro M.E.N.P., Vieira I.G.P., Gonalves T., Ricardo N.M.P.S., Fechine P.B.A. (2011). Magnetic nanoparticles for a new drug delivery systemto control quercetin releasing for cancer chemotherapy. J. Nanopart. Res..

[28] Flavin K., Resmini M. (2009). Imprinted nanomaterials: A new class of synthetic receptors. Anal. Bioanal. Chem..

[29] Spégel P., Schweitz L., Nilsson S. (2003). Selectivity toward multiple predetermined targets in nanoparticle capillary electrochromatography. Anal. Chem..

[30] Alvarez-Lorenzo C., Concheiro A. (2004). Molecularly imprinted polymers for drug delivery. J. Chromatogr. B.

[31] Ye L., Mosbach K. (2008). Molecular imprinting: Synthetic materials as substitutes for biological antibodies and receptors. Chem. Mater..

[32] Boos K.S., Fleischer C.T. (2001). Multidimensional on-line solid-phase extraction (SPE) using restricted access materials (RAM) in combination with molecular imprinted polymers (MIP). J. Anal. Chem..

[33] Gore M.A., Karmalkar R.N., Kulkarni M.G. (2004). Enhanced capacities and selectivities for cholesterol in aqueous media by molecular imprinting: Role of novel crosslinkers. J. Chromatogr. B.

[34] Pitarresi G., Pierro P., Giammona G., Iemma F., Muzzalupo R., Picci N. (2004). Drug release from *α*,*β*-poly(N-2-hydroxyethyl)-dl-aspartamide-based microparticles. Biomaterials.

[35] Puoci F., Cirillo G., Curcio M., Iemma F., Parisi O.I., Castiglione M., Picci N. (2008). Molecularly imprinted polymers for α-tocopherol delivery. Drug Deliv..

[36] Doak S.H., Griffiths S.M., Manshian B., Singh N., Williams P.M., Brown A.P., Jenkins G.J.S. (2009). Confounding experimental considerations in nanogenotoxicology. Mutagenesis.

[37] Liu Y., Nair M.G. (2010). An efficient and economical MTT assay for determining the antioxidant activity of plant natural product extracts and pure compounds. J. Nat. Prod..

[38] Priyadarsini R.V., Murugan R.S., Maitreyi S., Ramalingam K., Karunagaran D., Nagini S. (2012). The flavonoid quercetin induces cell cycle arrest and mitochondria-mediated apoptosis in human cervical cancer (HeLa) cells through p53 induction and NF-κB inhibition. Eur. J. Pharmacol..

[38] Jagtap S., Meganathan K., Wagh V., Winkler J., Hescheler J., Sachinidis A. (2009). Chemoprotective mechanism of the natural compounds, epigallocatechin-3-O-gallate, quercetin and curcumin against cancer and cardiovascular diseases. Curr. Med. Chem..

[39] AlaaEddeen M., Seufi A.M., Ibrahim S.S., Elmaghraby T.K., Hafez E.E. (2009). Preventive effect of the flavonoid, quercetin, on hepatic cancer in rats via oxidant/antioxidant activity: Molecular and histological evidences. J. Exp. Clin. Cancer Res..

[40] Boly R., Gras T., Lamkami T., Guissou P., Serteyn D., Kiss R., Dubois J. (2011). Quercetin inhibits a large panel of kinases implicated in cancer cell biology. Int. J. Oncol..

[41] Estella-Hermoso de Mendoza A., Préat V., Mollinedo F., Blanco-Prieto M.J. (2011). *In vitro* and *in vivo* efficacy of edelfosine-loaded lipid nanoparticles against glioma. J. Control. Release.

[42] Song X., Li J., Wang J., Chen L. (2009). Quercetin molecularly imprinted polymers: Preparation, recognition characteristics and properties as sorbent for solid phase extraction. Talanta.

